# Roles of aberrant hemichannel activities due to mutant connexin26 in the pathogenesis of KID syndrome

**DOI:** 10.1038/s41598-018-30757-3

**Published:** 2018-08-27

**Authors:** T. Taki, T. Takeichi, K. Sugiura, M. Akiyama

**Affiliations:** 1 Department of Dermatology, Nagoya University Graduate School of Medicine, 65 Tsurumai-cho, Showa-ku, Nagoya, Aichi 466-8550 Japan; 2 Department of Dermatology, Fujita Health University School of Medicine, 1-98 Dengakugakubo, Kutsukake-cho, Toyoake, Aichi 470-1192 Japan

## Abstract

Germline missense mutations in *GJB2* encoding connexin (Cx) 26 have been found in keratitis, ichthyosis and deafness (KID) syndrome. We explored the effects of three mouse Cx26 mutants (Cx26-G12R, -G45E and -D50N) corresponding to KID syndrome-causative human mutants on hemichannel activities leading to cell death and the expression of immune response-associated genes. We analyzed the 3D images of cells expressing wild-type (WT) or mutant Cx26 molecules to demonstrate clearly the intracellular localization of Cx26 mutants and hemichannel formation. High extracellular Ca^2+^ conditions lead to the closure of gap junction hemichannels in Cx26-G12R or Cx26-G45E expressing cells, resulting in prohibition of the Cx26 mutant-induced cell death. Fluorescent dye uptake assays revealed that cells with Cx26-D50N had aberrantly high hemichannel activities, which were abolished by a hemichannel blocker, carbenoxolone and 18α-Glycyrrhetinic acid. These results further support the idea that abnormal hemichannel activities play important roles in the pathogenesis of KID syndrome. Furthermore, we revealed that the expressions of *IL15*, *CCL5*, *IL1A*, *IL23R* and *TLR5* are down-regulated in keratinocytes expressing Cx26-D50N, suggesting that immune deficiency in KID syndrome expressing Cx26-D50N might be associated not only with skin barrier defects, but also with the down-regulated expression of immune response-related genes.

## Introduction

Keratitis, ichthyosis and deafness (KID) syndrome is named for its clinical triad of erythrokeratoderma, vascularizing keratitis and bilateral sensorineural hearing loss. The syndrome was first recognized as a distinct clinical entity by Skinner *et al*. in 1981^1^. Although KID syndrome is rare, the cutaneous and extracutaneous manifestations of the disease are profoundly serious and refractory to treatment^2^. Patients with KID syndrome often present with extensive skin involvement and are at high risk for cutaneous bacterial infection^3^. Nearly half of patients with KID syndrome have an episode of chronic skin infection^4^. Skin tumors, particularly trichilemmal tumors and squamous cell carcinoma (SCC), are common during the disease course^5^. Indeed, about 15% of KID syndrome patients are reported to develop SCC of the skin and the oral mucosa^6^. Germline missense mutations in *GJB2* encoding connexin (Cx) 26 have been found to be associated with KID syndrome^7^. Cxs are membrane proteins that are primarily involved in intercellular communication. They are synthesized in the endoplasmic reticulum (ER)-Golgi network, and six Cx molecules are oligomerized to form a connexon (hemichannel), which docks at cell–cell contact points to form a gap junction intercellular channel that allows exchanges of electrical signals and biochemically important molecules between neighboring cells. Hemichannels allow cells to communicate with the extracellular environment^8–13^. Although the causative genetic defect of KID syndrome has been identified^7^, the molecular mechanisms that lead to the skin phenotypes via dysfunction of gap junctions and/or aberrant functions of hemichannels are poorly understood^14^. Various experiments have shown that KID syndrome-causative *GJB2* mutations result in the formation of Cx26 hemichannels with aberrant activity^15–23^. However, the results of these experiments have not always been consistent. Even for an identical *GJB2* mutation, some reports have revealed that cell death is induced by the mutation, whereas others have ruled out cell death induction^18–20^. Some investigations have reported that the cell death was necrosis, whereas others have reported it was apoptosis^19,20^. It has been shown that elevated extracellular Ca^2+^ concentrations drive the hemichannels into their closed state^24^. However, a number of reports did not mention exact Ca^2+^ concentrations in their experiments. The present study characterizes the effects of three KID syndrome-causative *Gjb2* mutations (Cx26-G12R, -G45E and -D50N) on hemichannel activities, cell death and immune responses of the cells with information on the Ca^2+^ concentrations for each experiment. To more accurately elucidate the roles of mutant Cx26 proteins in KID syndrome pathogenesis, we analyzed the cells by three-dimensional (3D) imaging. In addition, dye uptake experiments reported in the literature employed hemichannel blockers, carbenoxolone (CBX) and flufenamic acid^16,25^. In the present study, we used 18α-Glycyrrhetinic acid (AGA) as an additional hemichannel blocker.

## Results

### Lethality of cells transfected with the *Gjb2* mutations Cx26-G12R or Cx26-G45E

To examine the effects of the KID syndrome-associated *Gjb2* mutations Cx26-G12R and -G45E on the intracellular localization of Cx26, HeLa (human cervical carcinoma) cells lacking endogenous gap junctions were transiently transfected with pIRES2-AcGFP1 Cx26-WT (wild-type), -G12R or -G45E-FLAG constructs (pIRES2-AcGFP1 *Gjb2* WT, c.34 G > C or c.134 G > A-FLAG constructs). In the first series of experiments, we incubated the cells in Dulbecco’s Modified Eagle Medium (DMEM) + fetal bovine serum (FBS), which contained 1.9 mM Ca^2+^, at transfection. The transfected cells were easily recognized by the presence of green fluorescence from enhanced green fluorescent protein (eGFP). Cells transfected with *Gjb2* c.34 G > C (Cx26-G12R) or c.134 G > A (Cx26-G45E) constructs started to detach from the culture slides at 48 h after transfection, and all the transfected cells died within approximately 3–4 days under the condition of 1.9 mM Ca^2+^ concentration. No gap junction plaques were observed between neighboring cells expressing Cx26-G12R or Cx26-G45E.

### Intracellular localization of Cx26-WT and Cx26-D50N mutant proteins

**Figure 1:**
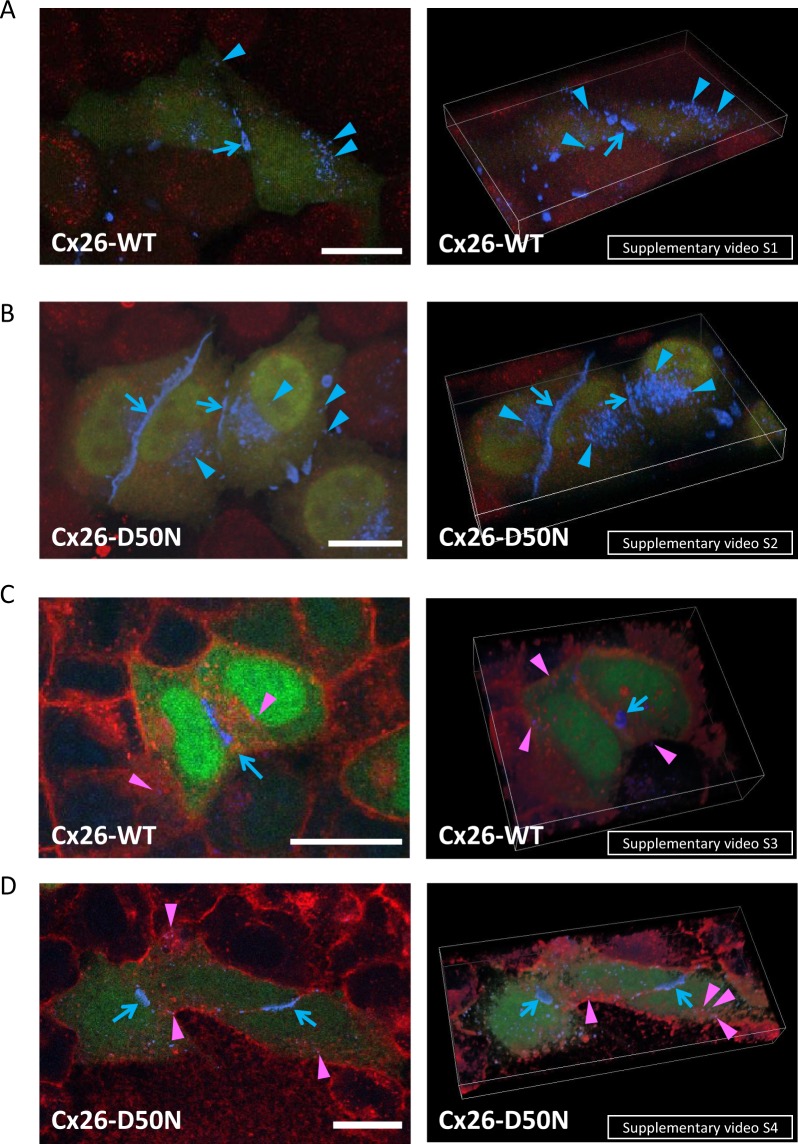
Formation of gap junctions and hemichannels with Cx26-WT or -D50N under the condition of 1.9 mM Ca^2+^ concentration. Left column: 2-dimensional images. Right column: 3-dimensional movies. (**A**) *Gjb2* WT-transfected HeLa cells were co-stained for TGN46, a marker for the trans-Golgi network (red), and for Cx26-WT-FLAG proteins (blue). The cells with green signals (eGFP) are the transfected cells. Blue arrows indicate gap junction plaques that formed between adjacent Cx26 expressing cells. Cx26-WT proteins are not localized in the trans-Golgi network marked with anti-TGN46 antibodies (red), and Cx26-WT blue dots may represent hemichannels (blue arrowheads) in the cell membrane. (**B**) *Gjb2* c.148 G > A-transfected HeLa cells were also co-stained for TGN46 (red) and Cx26-D50N-FLAG proteins (blue). Similarly to Cx26-WT, the mutant Cx26, Cx26-D50N, formed gap junctions at the cell-cell contact zones (blue arrows). Cx26-D50N is not observed in the trans-Golgi network and seems to form hemichannels in the cell periphery (blue arrowheads). (**C**) *Gjb2* WT-transfected cells were co-labeled with rhodamine-conjugated WGA (red) and Cx26-WT-FLAG (blue). The transfected cells show green signals (eGFP). Blue arrows indicate gap junction plaques connecting adjacent Cx26-WT-expressing cells. Purple signals (purple arrowheads) represent the co-localization of Cx26-WT and WGA, which are putative hemichannels. (**D**) *Gjb2* c.148 G > A-transfected cells were double-stained for rhodamine-labeled WGA (red) and Cx26-D50N-FLAG (blue). Gap junction formation (blue) is indicated with blue arrows. Purple signals (purple arrowheads) indicate the co-localization of Cx26-D50N and WGA, suggesting hemichannel formation in the cell surface. Scale bars: 20 μm.

We produced HeLa cells transiently transfected with pIRES2-AcGFP1 Cx26-WT constructs (pIRES2-AcGFP1 *Gjb2* WT-FLAG constructs) and HeLa cells transiently transfected with Cx26-D50N-FLAG constructs (pIRES2-AcGFP1 *Gjb2* c.148 G > A-FLAG constructs). We incubated the cells in DMEM + FBS, which contained 1.9 mM Ca^2+^, at transfection. Unlike the cells transfected with *Gjb2* c.34 G > C (Cx26-G12R) or c.134 G > A (Cx26-G45E), the HeLa cells with *Gjb2* WT (Cx26-WT) or c.148 G > A (Cx26-D50N) were able to proliferate even after transfection under the condition of 1.9 mM Ca^2+^ concentration. Immunofluorescent staining with ant-FLAG antibody (Cx26-FLAG staining) demonstrated that cells expressing Cx26-WT or Cx26-D50N were able to synthesize Cx26 proteins (Fig. 1, Supplementary Videos S1–S4). Further, Cx26 proteins were localized to the plasma membrane, and gap junction plaques were formed at the cell-to-cell contact zones between adjacent cells (Fig. 1, blue arrows, Supplementary Videos S1–S4). To clearly demonstrate the intracellular location of Cx26-WT and Cx26-D50N proteins, co-labeling with an anti-Cx26-FLAG antibody and an antibody to TGN46, which is a marker for the trans-Golgi network (TGN), or double staining with an anti-Cx26-FLAG antibody and wheat germ agglutinin (WGA) for plasma membrane staining were performed (Fig. 1 Supplementary Videos S1–S4). There was no overlap of Cx26 and TGN46 signals in cells expressing Cx26-WT or -D50N (Fig. 1A,B, blue arrowheads, Supplementary Videos S1 and S2). Furthermore, Cx26-FLAG and WGA co-staining showed that Cx26-FLAG staining overlapped with WGA staining in the cell membrane area without cell-to-cell contact and verified that Cx26-WT and -D50N were localized to the plasma membrane under the condition of 1.9 mM Ca^2+^ concentration (Fig. 1C,D, purple arrowheads, Supplementary Videos S3 and S4). In contrast, gap junction plaques showed no overlap of Cx26-FLAG with WGA (Fig. 1C,D, blue arrows, Supplementary Videos S3 and S4). WGA labels glycoproteins or glycolipids on the outer surface of the cell membrane^26^. Completely built gap junction plaques consist of a compact assembly of connexin molecules lacking surface glycoproteins and glycolipids. Thus, WGA cannot bind to the gap junction plaque. These findings clearly indicate that Cx26-WT and Cx26-D50N expressed in the HeLa cells were localized to the plasma membrane, but not to the Golgi apparatus, and that WGA was unable to access the gap junction plaques consisting of Cx26-WT and Cx26-D50N.

### High extracellular Ca^2+^ concentration rescues cells producing Cx26-G12R mutants and those producing Cx26-G45E mutants

**Figure 2:**
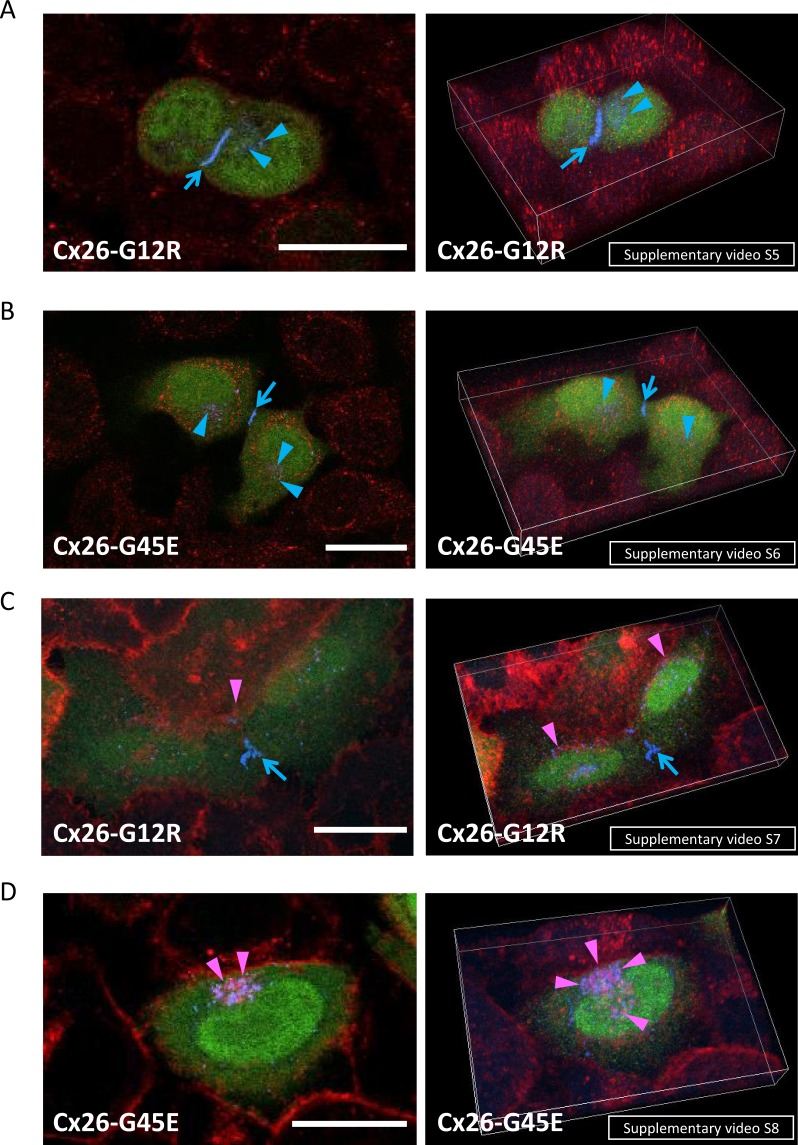
Formation of gap junctions and hemichannels with Cx26-G12R or Cx26-G45E under the condition of high extracellular Ca^2+^ concentration (3.8 mM Ca^2+^). Left column, 2-dimensional images. Right column, 3-dimensional movies. (**A**) The *Gjb2* c.34 G > C-transfected HeLa cells were co-stained for the trans-Golgi network marker TGN46 (red) and for Cx26-G12R-FLAG proteins (blue). The cells with green signals (eGFP) are transfected cells. Blue arrows indicate the gap junction plaques between adjacent Cx26-G12R expressing cells. Cx26-G12R proteins are not localized in the trans-Golgi network stained with anti-TGN46 antibodies (red), and Cx26-G12R blue dots (blue arrowheads) are putative hemichannels in the cell surface. (**B**) The *Gjb2* c.134 G > A-transfected HeLa cells were co-stained for TGN46 (red) and Cx26-G45E-FLAG proteins (blue). Cx26-G45E also formed gap junctions at the cell-cell contact zones (blue arrows). Blue dots of Cx26-G45E (blue arrowheads) are not seen in the trans-Golgi network (red). (**C**) *Gjb2* c.34 G > C-transfected cells were co-labeled with rhodamine-conjugated WGA (red) and Cx26-G12R-FLAG (blue). The transfected cells show green signals (eGFP). Small gap junction plaques (blue arrows) are seen between adjacent Cx26-G12R-expressing cells. Purple signals (purple arrowheads) represent the co-localization of Cx26-G12R and WGA, suggesting the formation of hemichannels in the cell surface. (**D**) The *Gjb2* c.134 G > A-transfected cells were double-stained for rhodamine-labeled WGA (red) and Cx26-G45E-FLAG (blue). Gap junction formation is not apparent. Purple signals (purple arrowheads) indicate the co-localization of Cx26-G45E and WGA, which are putative hemichannels in the cell surface. Scale bars: 20 μm.

We tested whether cell death due to Cx26-G12R mutants or Cx26-G45E mutants could be abolished by increased extracellular Ca^2+^ concentration (3.8 mM). The cell death induced by the *Gjb2* mutation Cx26-G12R and that induced by the *Gjb2* mutation Cx26-G45E were prohibited by the high extracellular Ca^2+^ concentration (3.8 mM). Cells transfected with Cx26-G12R or Cx26-G45E mutant DNA constructs showed no overlap of Cx26-FLAG and TGN46 (Fig. 2A,B, blue arrowheads, Supplementary Videos S5 and S6) under the condition of high extracellular Ca^2+^ concentration. Under the high extracellular Ca^2+^ condition, HeLa cells with Cx26-G12R or Cx26-G45E also formed gap junction plaques without WGA co-staining between adjacent cells (Fig. 2, blue arrows, Supplementary Videos S5–S8). Hemichannels co-stained with WGA were also detected (Fig. 2C,D, purple arrowheads, Supplementary Videos S7 and S8). These results suggest that both Cx26-G12R-expressing cells and Cx26-G45E-expressing cells were rescued by the high extracellular Ca^2+^ concentration and that the Cx26 proteins formed gap junction plaques and hemichannels in the rescued cells, similarly to gap junction plaques and hemichannels in the cells transfected with Cx26-WT or Cx26-D50N mutants. Consequently, cells carrying Cx26-G12R or Cx26-G45E were judged to be insufficiently healthy for further mutant protein localization studies and for gene expression profiling under the condition of physiological Ca^2+^ concentration.

### Altered activities of Cx26-D50N hemichannels

**Figure 3:**
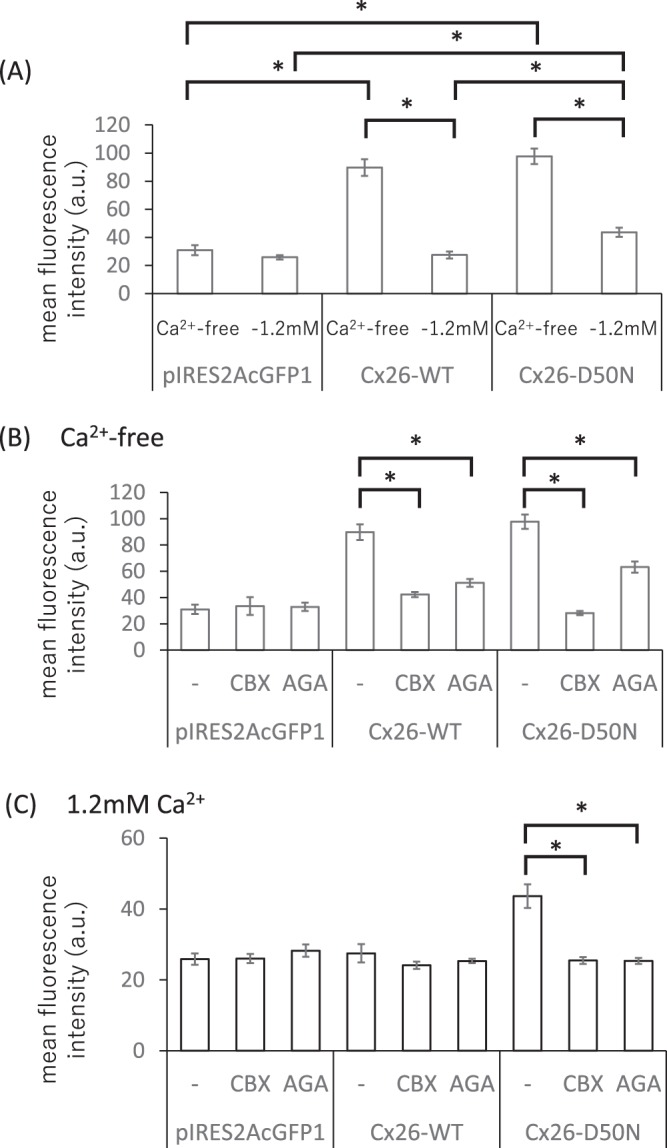
Fluorescent dye uptake in cells expressing Cx26-WT or Cx26-D50N. (**A**) Mean fluorescent dye intensities (levels of dye uptake) of cells expressing Cx26-WT or Cx26-D50N in the Ca^2+^-free medium and in the medium with physiological Ca^2+^ concentration (1.2 mM). The cells transfected with pIRES2AcGFP1 are negative control cells. Concerning both the cells expressing Cx26-WT and those expressing Cx26-D50N, the dye uptake levels of the cells are significantly lower (*p < 0.01) for cells in the physiological Ca^2+^ concentration condition than for cells in the Ca^2+^-free condition. Under the Ca^2+^-free condition, the dye uptake levels of the cells expressing Cx26-WT and the cells expressing Cx26-D50N are significantly higher (*p < 0.01) than those of the negative control cells. Under the physiological Ca^2+^ concentration condition, the dye uptake levels of the cells expressing Cx26-D50N are significantly higher (*p < 0.01) than those of the cells expressing Cx26-WT and the negative control cells. (**B**) Under the Ca^2+^-free condition, dye uptake levels of both the cells expressing Cx26-WT and those expressing Cx26-D50N significantly decreased (*p < 0.01) with the addition of hemichannel blockers, 100 μM CBX or AGA. (**C**) Under the physiological Ca^2+^ concentration condition, dye uptake levels of the cells expressing Cx26-WT are not high compared with those of the negative control cells. Dye uptake levels of the cells expressing Cx26-D50N significantly decreased (*p < 0.01) with the addition of hemichannel blockers, 100 μM CBX or AGA. The statistical significance between groups was assessed using paired Student’s *t*-test. Error bars represent the standard error of the mean. a.u.: arbitrary units.

We determined the effects of Cx26-D50N in mammalian cell lines with a fluorescent dye uptake assay using neurobiotin (NB) (Fig. 3). Under Ca^2+^-free conditions, the mean fluorescent intensity of NB in cells with Cx26-WT or Cx26-D50N hemichannels was three times as high as that in negative control cells (*p* < 0.01). Under the conditions of physiological Ca^2+^ concentration (1.2 mM), NB uptake was almost the same in cells with Cx26-WT as in the negative control cells, but NB uptake by Cx26-D50N-expressing cells was about 1.7 times as large as those by cells with Cx26-WT and negative control cells (*p* < 0.01) (Fig. 3A).

NB dye uptakes by the cells with Cx26-WT or Cx26-D50N under the condition of 1.2 mM Ca^2+^ concentration were less than those under the Ca^2+^-free conditions (*p* < 0.01). These results suggest that both normal hemichannels of Cx26-WT and aberrant hemichannels of Cx26-D50N had similar activities under the Ca^2+^-free conditions, but that aberrant hemichannel activities were aggravated under the condition of physiological Ca^2+^ concentration.

To further verify the formation of abnormal hemichannels in the plasma membrane, dye uptake studies were performed with the presence of hemichannel blockers, CBX and AGA (Fig. 3B,C). Under the Ca^2+^-free condition, the treatment of cells with 100 μM CBX or AGA for 20 min reduced the levels of dye uptake in both cells producing Cx26-WT and Cx26-D50N compared to their counterparts without CBX or AGA treatment, respectively (*p* < 0.01) (Fig. 3B). Under the condition of physiological Ca^2+^ concentration (1.2 mM), treatments of cells with 100 μM CBX or AGA for 20 min resulted in a 40% reduction in the levels of dye uptake (mean fluorescent intensities) in Cx26-D50N-expressing cells compared to their counterparts without CBX or AGA treatment, respectively (*p* < 0.01) (Fig. 3C). In contrast, cells producing Cx26-WT with or without hemichannel blocker treatment showed almost the same levels of dye uptake (mean fluorescent intensities). These findings suggest that the increase in the uptake of NB into cells was mediated by aberrant hemichannels consisting of Cx26-D50N.

### Cx26-D50N mutant down-regulates expression of genes involved in immune responses by keratinocytes

**Figure 4:**
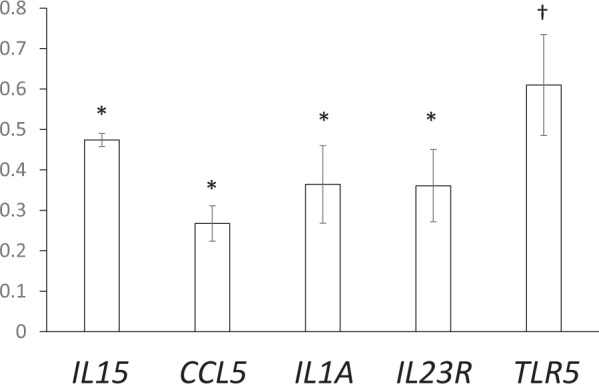
mRNA expression levels of the immune response-associated genes (*IL15*, *CCL5*, *IL1A*, *IL23R* and *TLR5*) by *Gjb2* c.148 G > A (Cx26-D50N)-transfected HaCaT cells. Relative mRNA expression levels of *Gjb2* WT-transfected HaCaT cells and *Gjb2* c.148 G > A (Cx26-D50N)-transfected cells were determined by real-time PCR. The results are expressed as the amount of mRNA normalized to GAPDH endogenous expression compared to control values (expression levels of *Gjb2* WT-transfected cells). mRNA expression levels of *IL15*, *CCL5*, *IL1A*, *IL23R* and *TLR5* were significantly decreased by the transfection of *Gjb2* c.148 G > A (Cx26-D50N) compared with the transfection of *Gjb2* WT (*p < 0.01 (n = 3); ^†^*p* < 0.05 (n = 3)). Data are presented as values calculated by the Delta-Delta-Ct (DDCt) method. The statistical significance between groups was assessed using paired Student’s *t*-test. Error bars represent the standard error of the mean.

To identify down-regulated genes in the HaCaT cells (human skin keratinocytes) producing Cx26-D50N, we analyzed data from gene expression profiling using the Clariom S array. Using a minimum fold change of 2.5, we selected 69 down-regulated genes. We evaluated these genes by using the functional annotation chart of DAVID Bioinformatics Resources 6.8 (https://david.ncifcrf.gov/). We initially chose 6 terms involved in immunological processes: “immunity”, “positive regulation of T cell proliferation”, “inflammatory response”, “innate immune response”, “TNF signaling pathway” and “defense response to bacterium”. Under these terms, we found 16 genes associated with immune function (IL23R, FCN1, CLEC4E, IFNL1, IFNL3, BIRC3, IL1A, IL15, USP41, CD180, TAPBPL, HMHB1, CCL5, TLR5, FCAMR and USP41) out of the 69 differentially expressed genes (16/69 genes, 23%). Next, we evaluated these genes by using the functional annotation clustering of DAVID Bioinformatics Resources 6.8. We adopted an enrichment score of more than 1.98 and an adjusted p-value of less than 0.05 to select these down-regulated genes, and we chose 9/16 genes involved in immunological processes. Among these genes, we selected a total of 5 genes (IL15, CCL5, IL1A, IL23R, and TLR5) that have been reported in numerous papers in the past. We confirmed the down-regulation of mRNA expression of those genes by real-time PCR (Fig. 4) and concluded that Cx26-D50N expression resulted in the down-regulated mRNA expression of *IL15*, *CCL5*, *IL1A*, *IL23R* and *TLR5*.

## Discussion

Elucidation of the effects of KID syndrome-causative Cx26 mutations might provide clues on how Cx26 functions in normal epidermal homeostasis and on the pathomechanisms of disease phenotypes in KID syndrome patients. In the present study, we examined how three *Gjb2* mutants (Cx26-G12R, -G45E and -D50N) causative of KID syndrome affected the formation and function of the hemichannels and gap junctions in transfected HeLa cells. In addition, we analyzed the cells by 3D imaging. In the 3D images, we are able to evaluate the molecular localization sites more accurately, although in 2D images we sometimes misunderstood the colocalization sites of target molecules.

*GJB2* mutations causative of KID syndrome have been shown to induce elevated hemichannel activities^22,27,28^. In the present study, the *Gjb2* mutations Cx26-G12R and Cx26-G45E led to increased hemichannel activity and induced cell death. The *Gjb2* mutation Cx26-D50N induced high hemichannel activity, but showed no cellular lethality. The cell death induced by the *Gjb2* mutation Cx26-G12R and that induced by the *Gjb2* mutation Cx26-G45E were rescued by the addition of extracellular Ca^2+^. It was previously shown that cells with *GJB2* mutations are rescued from cell death by the introduction of Ca^2+^ to the extracellular media during incubation, although cells carrying different *GJB2* mutations showed different reactions to high extracellular Ca^2+^ levels^28^. It is known that elevated extracellular Ca^2+^ drives the hemichannels into their closed state^24^. The high extracellular Ca^2+^ condition prohibited cell death induced by the *GJB2* mutant Cx26-G12R and that induced by the *GJB2* mutant Cx26-G45E^25,27,28^.

Regarding the hemichannel current, the cells carrying the *GJB2* mutation Cx26-G12R responded more quickly and completely to the high Ca^2+^ concentration switch than did the cells with the *GJB2* mutation Cx26-D50N^28^. Elevation of the external Ca^2+^ concentration reduced the amplitude of hemichannel currents by shifting the voltage activation curve of the channels to more positive potentials^29^. This indicates that different activation voltages resulting from the mutations might cause higher hemichannel activity leading to cellular lethality. The Cx26 mutant Cx26-D50N was previously reported to induce elevated membrane currents in *Xenopus* oocytes as measured electrophysiologically^30^. Cells expressing the Cx26-D50N showed abnormal hemichannel activity and increased cell death in the absence of elevated extracellular Ca^2+^^28^. Recently, several studies have used highly sophisticated methodologies to investigate the calcium gating of Cx26-D50N hemichannels. Lopez *et al.*^30–32^ reported that extracellular Ca^2+^ destabilizes the open state of human Cx26 hemichannels by disrupting a salt bridge interaction between residues D50 and K61 located close to the extracellular entrance of the pore of the hemichannels. This open-state destabilization is thought to facilitate hemichannel closure^30,31^. A highly conserved electrostatic network located at the extracellular entrance of the pore is involved in the gating of hemichannels of all connexin types. Extracellular Ca^2+^ disrupts the open-channel form of this network, resulting in a closed conformation^32^. Another study by Helmuth *et al*.^33^ revealed that Q48 and D50 tightly interact and that disruption of this interaction activates hemichannel gating along the voltage axis. Further shifts in gating activation by extracellular Ca^2+^ persist even in the absence of the Q48-D50 interaction, but the shifts require an Asp (D) or Glu (E) at position 50^33^. This fact suggests an independent electrostatic mechanism of gate activation that significantly involves position 50. The difference of Cx26-D50N from Cx26-G12R and Cx26-G45E in terms of their *in vivo* and *in vitro* effects may due to the unique characteristics of D50. In the present study, hemichannel activity in Cx26-D50N was less responsive to changes in the extracellular Ca^2+^ concentration. Summarizing the results of hemichannel activities in the present study, we clearly demonstrated the formation of aberrant hemichannels by the KID syndrome-causative *Gjb2* mutations Cx26-G12R, -G45E and -D50N. The present results further support the idea that abnormal hemichannel activities play an important role(s) in the pathogenesis of KID syndrome.

Hearing impairment-associated Cx26 mutants are roughly classified into two categories. Cx26 mutants in the first category are still localized to the cell membrane and form non-functional gap junctions. Cx26 mutants in the second category accumulate in the cytoplasm due to the abnormal trafficking^25^. The Cx26 mutants in this category localize within subcellular structures, including the ER, the ER-Golgi intermediate compartment and the Golgi apparatus^34–37^. In the present study, it was difficult to clarify the relationship between the Cx26 mutants and the trans-Golgi network. However, in the 3D images, none of the Cx26 mutants showed overlapping localization with TGN46 protein signals under the condition of high extracellular Ca^2+^ levels. This indicates that the *Gjb2* mutants Cx26-G12R, -G45E and -D50N can be classified into the first category. Most of the mutations associated with KID syndrome have shown to cause aberrant hemichannel activities, leading to the altered regulation of molecular exchanges through the plasma membrane^22,25,27,28^. As is true for the many characterized cell lines from KID syndrome patients^16,25^, in the present study, cells with Cx26 constructs with the mutation Cx26-D50N showed increased uptake of NB fluorescent dye, and the increased uptake was abolished by the hemichannel blockers CBX and AGA. This suggests that aberrant hemichannel activities are involved in the pathomechanisms of KID syndrome phenotypes due to the Cx26-D50N mutation.

Patients with KID syndrome are at high risk for neoplastic complications or cutaneous infections such as those of *Candida albicans* and *Trichophyton rubrum*^6^. Although the genetic defect of KID syndrome has been identified, the mechanisms behind the high incidence of neoplastic complications and fungal infections in KID syndrome are poorly understood. Increased risk of malignant tumors and chronic infections in KID syndrome is likely attributable to impaired epithelial barrier function or defective immune function^38^. Regarding immune function in KID syndrome patients, some studies have reported it to be normal^39,40^, but others have showed immune dysregulation^41^ or have suggested primary systemic immunodeficiency^3,42^. In the present study, we determined that human keratinocytes expressing the Cx26-D50N mutant show down-regulated expression of *IL15*, *CCL5*, *IL1A*, *IL23R* and *TLR5*, compared with keratinocytes expressing Cx26-WT. Interleukin-15 (IL-15) exhibits biological activities similar to those of interleukin-2 and enhances the proliferation of CD8^+^ cytotoxic T cells and natural killer cells, which in turn eliminate tumor cells^43–49^. Chemokine C-C motif ligand 5 (CCL5) plays an important role in recruiting various leukocytes, including T cells, macrophages, eosinophils, and basophils, to inflammatory sites. In collaboration with certain cytokines released from T cells, CCL5 also activates natural killer cells to C-C chemokine-activated killer cell and induces their proliferation^50^. Interleukin-1α (IL1α) is produced by cells of various types, including activated macrophages, keratinocytes, stimulated B lymphocytes, and fibroblasts, and it is a potent mediator of inflammation and immune reactions^51^. In humans, high levels of Interleukin-23 receptor (IL-23R) expression are detected on activated/memory T cells, NK cells, macrophages, dendritic cells and monocytes. IL-23 and Interleukin-12 (IL-12) share a common subunit in their receptor complex. The receptor complex is a heterodimer made up of 2 subunits, IL-23R and IL-12β1^52^. Stimulation of the receptor complex activates janus activating kinase 2 (JAK2) and tyrosine kinase 2 (TYK2), resulting in phosphorylation of the receptor complex, and the formation of docking sites for signal transducers and activators of transcription (STATs) 1, 3, 4 and 5. The STATs are subsequently dimerized, phosphorylated and translocated into the nucleus, activating target genes^53^. Importantly, the phosphorylation of STAT4 is essential for increasing interferon (IFN) γ production and the subsequent differentiation of Th1 cells, whereas STAT3 is important for the development of interleukin 17-producing helper T cells (Th17 cells)^54,55^. Overall, this process orchestrates the cytokine cascade, activating the necessary immune cells involved in the eradication of any pathogenic/antigenic challenge.

Toll-like receptors (TLRs) provide efficient and immediate immune responses to bacterial, fungal, and viral infections by recognizing diverse molecules released from them^56^. One study reports that an Asian KID syndrome patient with fungal infection expressed only a lower level of TLR2 mRNA^57^. The present results clearly indicate that keratinocytes expressing the Cx26-D50N mutant show down-regulated expression of important molecules involved in the epidermal immune responses including IL-15, CCL5, IL-1α, IL-23 receptor and TLR5. We speculate that the down-regulation might cause immunodeficiency in the epidermis of KID syndrome due to the Cx26-D50N mutant, resulting in the malignant tumors and chronic infections seen in the patients.

Observations of 2-dimensional plane images are insufficient to clarify the intracellular localization of Cx26 proteins and the formation sites of hemichannels. In the present study, we analyzed the 3D images of cells expressing WT and mutant Cx26 molecules and were able to demonstrate clearly the intracellular localization of Cx26 proteins and hemichannel formation sites.

In conclusion, we clearly demonstrated that aberrant hemichannels form due to the KID syndrome-causative *Gjb2* mutations Cx26-G12R, -G45E and -D50N. Our findings further support the idea that abnormal hemichannel activities are involved in the pathogenesis of KID syndrome. In addition, we revealed that the expressions of *IL15*, *CCL5*, *IL1A*, *IL23R* and *TLR5* are down-regulated in keratinocytes expressing the Cx26-D50N mutation. These findings suggest that the immune deficiency of KID syndrome caused by Cx26-D50N mutation owes not only to skin barrier dysfunction, but also to the down-regulated expression of immune response-related genes in the keratinocytes.

## Methods

### Cell culture

Parental HeLa cells were grown as previously described^58^. The keratinocytes cell line HaCaT was kindly provided by N Fusenig (German Cancer Research Center, Heidelberg, Germany)^59^. HeLa cells that have no gap junction communication channel, and HaCaT cells were maintained in DMEM (Thermo Fisher Scientific, Waltham, Massachusetts, USA) supplemented with 10% FBS (Equitech-Bio Inc., Kerrville, Texas, USA) in a humidified chamber with 5% CO_2_ at 37 °C. This DMEM + FBS contained 2.0 mM Ca^2+^.

### Construction of mouse *Gjb2* mutant clones

The *Gjb2* cDNA constructs pCMV-*Gjb2* (WT, c.34 G > C, c.134 G > A and c.148 G > A)-FLAG-IRES2-AcGFP1 (Takara Bio Inc., Shiga, Japan) were produced as follows: *Gjb2* (WT, c.34 G > C, c.134 G > A or c.148 G > A)-FLAG genes were synthesized artificially and subcloned into the Nhel/BamHI site in the pIRES2-AcGFP1 vector (Clontech, Mountain View, California, USA). The constructs were used for transfection experiments including immunocytochemical, dye uptake, microarray and functional studies targeting HeLa cells, which do not have any endogenous gap junctions. If products of transfected KID syndrome-causative mutations coexisted with other endogenous connexins, we were afraid that such coexistence might affect the interaction and functional properties of mutant Cx26^17^. We selected mouse *Gjb2* mutations to minimize the influence of human endogenous Cxs.

### Transfection experiments targeting HeLa Cells

Briefly, 1 day before transfection, cells were plated in 8-well glass slides (Thermo Fisher Science, Waltham, Massachusetts, USA) in DMEM + FBS (225 μl, Ca^2+^ concentration, 2.0 mM) and they were 70–95% confluent on the day of transfection. Lipofectamine 3000 reagent, plasmids, and OPTI-MEM medium (Life Technologies, California, USA) were brought to room temperature. DNA (total of 0.1 μg), Lipofectamine 3000 reagent (0.4 μl) and P3000 reagent (0.2 μl) were diluted in 5 μl of OPTI-MEM following the manufacturer’s protocol. Following 15 min of incubation at room temperature, the DNA, Lipofectamine and OPTI-MEM mixture (10 μl, Ca^2+^ concentration, 0 mM) was added to the cells drop by drop, and the cells were incubated at 37 °C for 48 h in the DMEM + FBS with DNA, Lipofectamine and OPTI-MEM mixture (Ca^2+^ concentration, 1.9 mM). The cells were then used for immunofluorescence staining or dye uptake assays.

### Immunofluorescence staining

HeLa cells (2.0 × 10^4^) were grown over glass coverslips in 8-well glass slides for immunofluorescent staining experiments. At 48 h post-transfection, the cells were fixed with 4% paraformaldehyde (PFA) for 15 min, washed twice with phosphate buffered saline (PBS), permeabilized with 0.25% Triton-X 100 (Wako, Tokyo, Japan) for 30 min, washed twice with PBS and blocked with 10% bovine serum albumin (BSA) for 30 min at 37 °C. The cells were then incubated overnight with M2 monoclonal mouse antibody against FLAG (Sigma-Aldrich, St. Louis, USA) diluted to 1:1000 at RT, washed twice with PBS followed by the application of tetra-methyl rhodamine isothiocyanate (TRITC)-conjugated rabbit polyclonal anti-mouse immunoglobulin antibody (Dako Cytomation, Glostrup, Denmark), diluted to 1:1000 and left for 45 min at 37 °C in the dark. After two more washings with PBS, coverslips were mounted with VECTORSHIELD mounting medium with DAPI (Vector laboratories, California, USA) on glass slides.

For co-immunofluorescent staining for TGN46, which is a marker for the trans-Golgi network, and for Cx26-FLAG proteins, the cells were incubated with primary antibodies (M2 monoclonal mouse antibody against FLAG (1:1000) and rabbit anti-TGN46 (1:500) (Abcam, Cambridge, UK)) for 2 h at RT. After two more washings with PBS, secondary antibodies (goat polyclonal anti-mouse IgG antibody, Alexa Flour 350 (1:1000) (Invitrogen, Massachusetts, USA) and TRITC-conjugated swine polyclonal anti-rabbit immunoglobulins antibody (1:1000) (Dako Cytomation, Glostrup, Denmark)) were applied for 45 min at 37 °C in the dark. After two more washings with PBS, coverslips were mounted with VECTORSHIELD mounting medium (Vector laboratories, California, USA) on glass slides.

For co-labeling for rhodamine-labeled WGA and Cx26-FLAG, after blocking with 10% BSA, rhodamine-labeled WGA (5 mg/ml, Vector laboratories, California, USA) diluted 1:500 was applied to cells for 30 min at 37 °C. After two washings with PBS, the cells were incubated with primary antibodies (M2 monoclonal mouse antibody against FLAG (1:1000)) overnight at RT. After two more washings with PBS, secondary antibodies (goat polyclonal anti-mouse IgG antibody, Alexa Flour 350 (1:1000) (Invitrogen, Massachusetts, USA)) were applied for 45 min at 37 °C in the dark. After two more washings with PBS, coverslips were mounted with VECTORSHIELD mounting medium on glass slides.

Staining was verified using an A1R confocal laser scanning microscope system, TiE-A1R, (Nikon, Japan) with x40 and x100 oil-immersion objectives.

### Dye uptake assays

HeLa cells (2.0 × 10^4^) were grown over glass coverslips in 8-well glass slides for dye uptake assays with an NB tracer (NB, FW 322.8, Vector Laboratories). At 48 h post-transfection (Ca^2+^ concentration, 1.9 mM), the cells were washed with PBS and incubated with Ca^2+^-free medium or a medium with a physiological calcium concentration (1.2 mM Ca^2+^) for 20 min at 37 °C. The cells were then incubated with 0.5 mg/ml NB in the same Ca^2+^ concentration medium for 20 min at 37 °C^22^. Next, the cells were washed with DMEM containing 4.0 mM CaCl_2_ twice for 10 min and PBS once. The cells were fixed with 4% PFA for 15 min at room temperature. After being washed twice with PBS, the fixed cells were permeabilized with 0.25% Triton-X 100 for 30 min, washed twice with PBS, and blocked with 10% BSA for 30 min at 37 °C. Following the two washings with PBS, the cells were incubated with primary antibodies (mouse monoclonal anti-FLAGR M2 antibody (1:1000)) overnight at RT. After two more washings with PBS, TRITC-conjugated rabbit polyclonal anti-mouse immunoglobulins antibody (1:1000) and Alaxa Flour 350-conjugated Streptavidin (1:200) (Invitrogen, Massachusetts, USA) were applied for 45 min at 37 °C in the dark. After two more washings with PBS, images were acquired using the A1R confocal laser scanning microscope system with fixed exposure times. For treatments with carbenoxolone (CBX, Sigma-Aldrich, St Louis, USA) or 18α-Glycyrrhetinic acid (AGA, Sigma-Aldrich), the cells were initially incubated with calcium-free medium containing 100 μM CBX or AGA for 20 min, and then NB in the same medium was applied to the cells as described above.

Image analysis for signal intensity determination was performed with ImageJ software (NIH, Bethesda, USA). We selected 2 high-power view fields under the microscope. During image analysis, after the background in merged images of red and green channels was subtracted, the same parameters were applied to threshold the images for the measurement of blue signal intensities of fluorescence only in the GFP-positive cells^16^. Each experiment was performed at least twice.

### Transfection experiments targeting HaCaT cells for microarray analysis

HaCaT cells were transfected using the Amaxa Cell Line Nucleofector Kit V (Lonza, Cologne, Germany) according to the manufacturer’s instructions. The cells were seeded on the plate and the medium was changed 3 h after seeding. At 8 h of incubation, the cells were subjected to microarray analysis and quantitative real-time reverse transcription (qRT)-PCR assays.

### Microarray analysis

We isolated total RNA from the transfected HaCaT cells using the RNeasy Mini Kit (Qiagen, Hiden Germany). Whole-genome expression profiling was performed using Clariom S Array (Affymetrix, Santa Clara, California, USA). Differentially expressed genes were defined as those showing a 2.5-fold or greater change in expression between the cells transfected with *Gjb2* WT constructs (Cx26-WT) and those transfected with the c.134 G > A constructs (Cx26-D50N) using the Affymetrix Transcriptome Analysis Console Ver 3.1.0.5 (Affymetrix). The list of down-regulated genes was analyzed using gene-set enrichment analyses from DAVID Bioinformatics Resources 6.8 (https://david.ncifcrf.gov/) in order to identify the functions of these genes. We used an enrichment score of more than 1.9 and an adjusted p-value of less than 0.05 to select these down-regulated genes.

### qRT-PCR

We isolated total RNA from the transfected HaCaT cells using the RNeasy Mini Kit (Qiagen). We reverse-transcribed 250 ng total RNA using the Prime Script RT Reagent Kit (Takara, Shiga, Japan) according to the manufacturer’s instructions. The recovered cDNA was diluted 10-fold with DW for qRT-PCR. mRNA expression levels were measured by qRT-PCR using the Light Cycler System (Roche, Basel, Switzerland). The PCRs were set up in microcapillary tubes filled with 10 μL of reaction agents including 2.5 μL of diluted cDNA solution, and the PCR program was set according to the manufacturer’s instructions. The primers and probes used for qRT-PCR are listed in Supplementary Table 1. Each experiment was performed at least three times.

### Statistical analyses

All results are expressed as mean (± standard deviation). In the dye uptake assay, we selected 2 high-power view fields under the microscope without any random sampling method and each experiment was performed at least twice. In the qRT-PCR analysis, each experiment was performed at least three times. Analysis of dye uptake samples and qRT-PCR samples was done by Student’s t-test. Statistical significance was shown as *p < 0.01, ^†^*p* < 0.05.

## Electronic supplementary material

Supplementary Video S1

Supplementary Video S2

Supplementary Video S3

Supplementary Video S4

Supplementary Video S5

Supplementary Video S6

Supplementary Video S7

Supplementary Video S8

Supplementary table1
