# Supplementary material for: Roles of aberrant hemichannel activities due to mutant connexin26 in the pathogenesis of KID syndrome
Source: Sci Rep. 2018 Aug 27;8:12824. doi: 10.1038/s41598-018-30757-3 (PMC6110719; doi:10.1038/s41598-018-30757-3)
Supplement: Supplementary file 9 — Supplementary table1 [file 41598_2018_30757_MOESM9_ESM.docx]

**Title**

**Roles of aberrant hemichannel activities due to mutant connexin26 in the pathogenesis of KID syndrome**

T. Taki^1^, T. Takeichi ^1^, K. Sugiura^2^, M. Akiyama^1,*^

^1^Department of Dermatology, Nagoya University Graduate School of Medicine, 65 Tsurumai-cho, Showa-ku, Nagoya, Aichi 466-8550, Japan

^2^Department of Dermatology, Fujita Health University School of Medicine, 1-98 Dengakugakubo, Kutsukake-cho, Toyoake, Aichi 470-1192, Japan

**Supplementary information**

**Supplementary Table 1**

The Taqman Gene Expression probes used for qRT-PCR

| Gene |  |  |
| --- | --- | --- |
| Separated probes |  | Nucleotide sequences |
| *IL15* | Forward Primer | AACTGAAGCTGGCATTCATGTC |
| NM_000585 | Reverse Primer | ACTTATTACATTCACCCAGTTGGC |
|  | Probe | AGGAAGCCCTGCACTGAAACAGCC |
| *CCL5* | Forward Primer | CATCTGCCTCCCCATATTCCTC |
| NM_002985 | Reverse Primer | CTGGTGTAGAAATACTCCTTGATGTG |
|  | Probe | CACCACACCCTGCTGCTTTGCCTACATT |
| *IL1A* | Forward Primer | AATCCATCACTGATGATGAC |
| NM_000575 | Reverse Primer | TTGTATTTCACATTGCTCAG |
|  | Probe | CATCGCCAATGACTCAGAGGAAG |
| Gene |  |  |
| Premixed probes | Assay ID | Dye label |
| *IL23* | Hs00332759_m1 | FAM_MGB |
| *TLR5* | Hs01920773_s1 | FAM_MGB |
| *GAPDH* | Hs02758991_g1 | VIC_PL |
